# The renin–angiotensin system: a possible new target for depression

**DOI:** 10.1186/s12916-017-0916-3

**Published:** 2017-08-01

**Authors:** João Vian, Círia Pereira, Victor Chavarria, Cristiano Köhler, Brendon Stubbs, João Quevedo, Sung-Wan Kim, André F. Carvalho, Michael Berk, Brisa S. Fernandes

**Affiliations:** 1 Psychiatry and Mental Health Department, Centro Hospitalar Lisboa Norte, Lisbon, Portugal; 2 Faculdade de Medicina da Universidade de Lisboa, Lisbon, Portugal; 3 Institut de Neuropsiquiatria i Adiccions (INAD), Parc de Salut Mar (PSM), Barcelona, Spain; 4 Translational Psychiatry Research Group and Department of Clinical Medicine, Faculty of Medicine, Federal University of Ceará, Fortaleza, Brazil; 5 Health, Social Care and Education, Anglia Ruskin University, Chelmsford, UK; 6 Institute of Psychiatry, Psychology and Neuroscience (IoPPN), King’s College London, London, UK; 7 Institute of clinical Research and Education in Medicine (IREM), Padova, Italy; 8 Physiotherapy Department, South London and Maudsley NHS Foundation Trust, Denmark Hill, London, SE5 8AZ UK; 9 Translational Psychiatry Program, Department of Psychiatry and Behavioral Sciences, McGovern Medical School, The University of Texas Health Science Center at Houston (UTHealth), Houston, TX USA; 10 Center of Excellence on Mood Disorders, Department of Psychiatry and Behavioral Sciences, McGovern Medical School, The University of Texas Health Science Center at Houston (UTHealth), Houston, TX USA; 11 Neuroscience Graduate Program, The University of Texas MD Anderson Cancer Center UTHealth Graduate School of Biomedical Sciences, Houston, TX USA; 12 Laboratory of Neurosciences, Graduate Program in Health Sciences, Health Sciences Unit, University of Southern Santa Catarina (UNESC), Criciúma, SC Brazil; 13 Deakin University, IMPACT Strategic Research Centre, School of Medicine, University Hospital Geelong, Barwon Health, Geelong, VIC Australia; 14 Department of Psychiatry, Orygen the National Centre of Excellence for Youth Mental Health and Orygen Research Centre, and the Florey Institute for Neuroscience and Mental Health, University of Melbourne, Melbourne, VIC Australia; 15 Department of Psychiatry, Chonnam National University Medical School, Gwangju, Republic of Korea

**Keywords:** Depression, Psychiatry, Inflammation, Renin–angiotensin system, Angiotensin, ATR1, ATR2, Mas, Angiotensin receptor blockers, Angiotensin-converting enzyme inhibitors

## Abstract

Depression remains a debilitating condition with an uncertain aetiology. Recently, attention has been given to the renin–angiotensin system. In the central nervous system, angiotensin II may be important in multiple pathways related to neurodevelopment and regulation of the stress response. Studies of drugs targeting the renin–angiotensin system have yielded promising results. Here, we review the potential beneficial effects of angiotensin blockers in depression and their mechanisms of action. Drugs blocking the angiotensin system have efficacy in several animal models of depression. While no randomised clinical trials were found, case reports and observational studies showed that angiotensin-converting enzyme inhibitors or angiotensin receptor blockers had positive effects on depression, whereas other antihypertensive agents did not. Drugs targeting the renin–angiotensin system act on inflammatory pathways implicated in depression. Both preclinical and clinical data suggest that these drugs possess antidepressant properties. In light of these results, angiotensin system-blocking agents offer new horizons in mood disorder treatment.

## Background

The pathophysiology of depression remains elusive and current treatments, which focus on traditional pathways (monoamine alterations), are only partially effective. Remission rates in the treatment of depression are only about 30% for those treated with traditional pharmacotherapy, and multiple agents are often required to achieve an adequate level of recovery [1] Evidence points to the involvement of neuroinflammation, oxidative and nitrosative stress pathways, mitochondrial dysfunction and neurotrophic signalling in depression [2].

Recently, the renin–angiotensin system (RAS) was proposed to be implicated in depression, and that blocking this system, either with angiotensin-converting enzyme inhibitors (ACEIs) or with angiotensin II type 1 receptor (AT1R) blockers, would translate into clinical benefits for the depression treatment [3–7]. Here, we review the literature so far on RAS-targeting drugs in depression.

## Methods

A PubMed search was conducted for literature published between January 1974 and June 2017. Search terms included were: depression OR inflammation OR anxiety OR mood AND renin–angiotensin system, angiotensin, ATR1, ATR2, angiotensin receptor blockers, angiotensin-converting enzyme inhibitors, ATR3, ATR4, Mas, and aldosterone. Systematic reviews, randomised controlled trials (RCTs), observational studies, case series and animal studies with an emphasis on the angiotensin system and its role in depression were included. Articles not in English were excluded. The PubMed search was augmented by manually searching the references of key papers and related literature. The results were presented as a narrative review.

## The RAS in the brain

The RAS was discovered in the 19^th^ Century, after the blood pressure-raising agent renin was first identified in the rabbit kidney [8]. In time, the RAS became an established and extensively studied peripheral regulator of blood pressure and renal-mediated body fluid homeostasis, and was discovered to be a central target in clinical hypertension therapy. Renin, a protein synthesised by the juxtaglomerular cells of the kidney, cleaves the polypeptide angiotensinogen to generate angiotensin I (Ang I). This peptide is metabolised to angiotensin II (Ang II) by angiotensin I-converting enzyme (ACE).

It was surprising when renin was identified in the dog brain in 1971 [9, 10]. Subsequently, intracranial Ang II was shown to elevate blood pressure and to promote fluid intake [11–14], suggesting that angiotensin receptors were present in the brain. The actions of Ang II in the central nervous system are mediated mainly by two receptor types: AT1R and AT2R [15, 16]. Other receptors, including MAS [17], the (pro)renin receptor (PRR) [18] and AT4R [19], have also recently been identified but their roles remain less well characterised. AT3R was first reported as a new binding site for Ang II in mouse neuroblastoma cell cultures [20], but a separate gene for this receptor remains to be sequenced in humans.

**Fig. 1:**
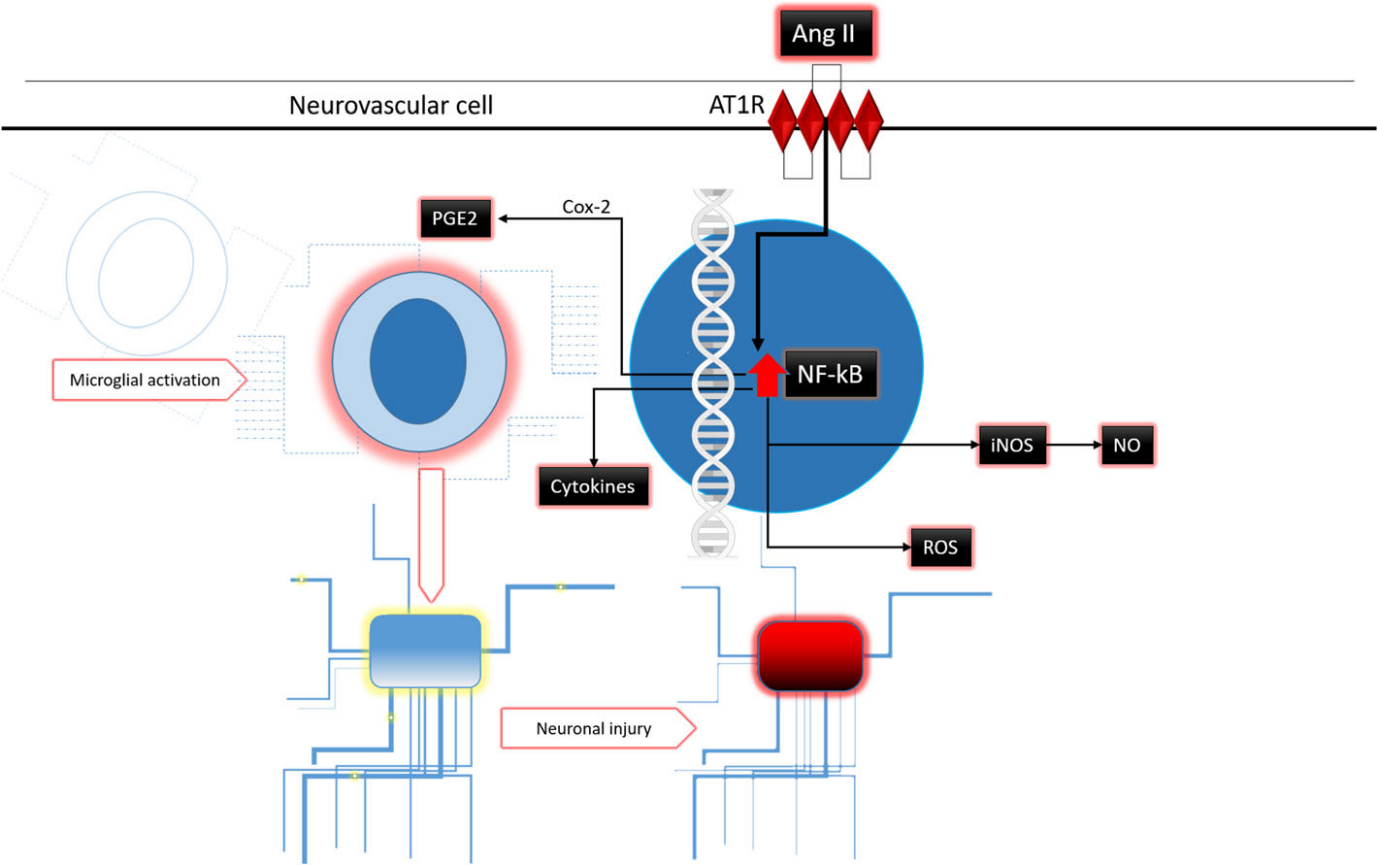
Pathways involved in neuronal damage of angiotensin II through AT1 receptor agonism. Ang II, angiotensin II; AT1R, angiotensin II receptor type 1; PGE2, prostaglandin E2; Cox-2, Cyclooxygenase-2; PPAR-γ, peroxisome proliferator-activated receptor gamma; NF-kB, nuclear factor kappa-light-chain-enhancer of activated B cells; iNOS, inducible nitric oxide synthase; NO, nitric oxide; ROS, reactive oxygen species

AT1R mediates most of the peripheral and central actions of Ang II [21] and is implicated in multiple pathways related to regulation of the stress response. Stimulating AT1R contributes to the release of inflammatory markers [22]. Ang II interacts with AT1Rs, activating the NADPH–oxidase complex [23–25], the microglial RhoA/Rho kinase pathway [26–28], NF-kappa B, inducible nitric oxide synthase (iNOS) and cyclooxygenase-2 (COX-2). In turn, activated COX-2 forms an intermediate in several key aspects of central nervous system inflammation, and in oxidative and nitrosative stress (see Fig. 1). AT1R stimulation also releases tumour necrosis factor α (TNF-α) [29, 30], which is important in several neurodegenerative disorders [29, 31–33], and regulates activation of the hypothalamic–pituitary–adrenal axis. Stimulation of AT1R in the parvocellular hypothalamic paraventricular nucleus (PVN) by Ang II increases production of corticotrophin-releasing factor [34–36]. In turn, this spurs adrenocorticotropic hormone secretion in the anterior pituitary gland, starting the stress response cascade. Accordingly, in humans, AT1R blockade downregulates hypothalamic–pituitary–adrenal axis activation [37].

Ang II also stimulates the release of aldosterone via AT1R in the adrenal cortex of the kidney [38]. Thus, the acronym ‘RAAS’ (as in renin–angiotensin–aldosterone system) is often used. Besides being regulated by Ang II, aldosterone release is also stimulated by adrenocorticotropic hormone and the sympathetic nervous system. The role of aldosterone in the brain has previously been downplayed because its specific intracellular receptor, the mineralocorticoid receptor (MR), shares affinity with cortisol, which circulates at a ~1000-fold higher concentration than aldosterone [39]. For a tissue to be sensitive to aldosterone, it must express 11β-hydroxysteroid dehydrogenase type 2 (HSD-2) protein, which degrades cortisol, freeing the MR to the action of aldosterone. HSD-2 has been identified in the brain, mainly in the nucleus of the solitary tract, but also in the PVN [40]; regions that also express AT1R. Surprisingly – paralleling the history of angiotensin – aldosterone synthesis was also recognised in the amygdala, hippocampus and hypothalamus of the brain [41].

AT1R is particularly dense in the anterior pituitary; the circumventricular organs (area postrema; subfornical organ, the vascular organ of lamina terminalis and the median eminence); the lateral geniculate body; inferior olivary nucleus; the nucleus of the solitary tract and in the PVN, the preoptic and the supraoptic nuclei of the hypothalamus [42].

Modern molecular approaches have revealed that AT2R is also expressed in the adult brain [43, 44]. AT2R is involved in neurodevelopment [45–49] and participates in cell growth inhibition, fetal tissue development, extracellular matrix modulation, neuronal regeneration, apoptosis, cellular differentiation, and, possibly, vasodilation and left ventricular hypertrophy [50]. AT2R stimulation exerts neuroprotective effects in ischaemic stroke in rodents [51–55], and while the underlying mechanism remains to be fully characterised, it seems to partly involve an increase in the anti-inflammatory cytokine interleukin-10 [56]. AT2R is particularly dense in the amygdala, caudate putamen, medial geniculate body, globus pallidus, habenula, hypoglossal nucleus, inferior colliculus, inferior olivary nucleus, locus coeruleus, thalamus, and ventral tegmental area [42].

**Fig. 2:**
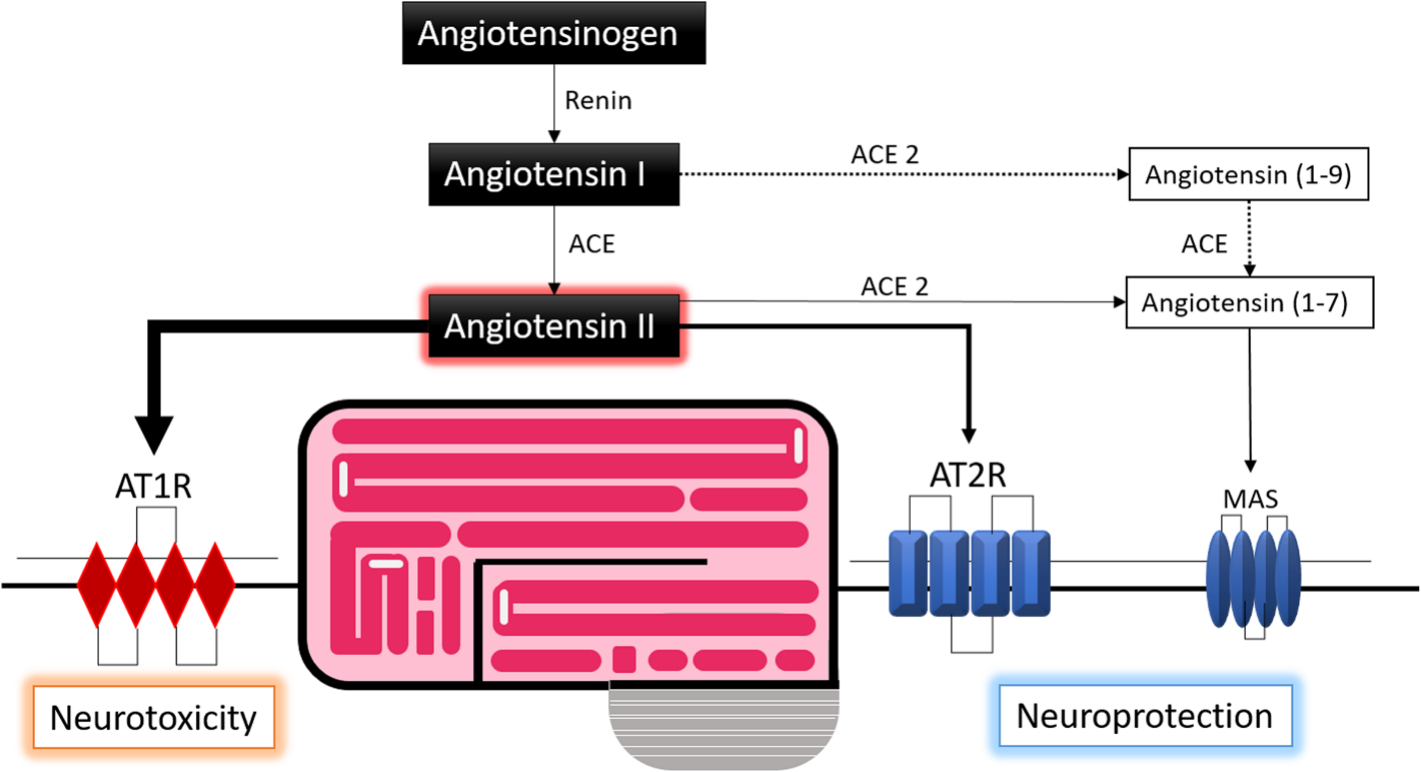
Pathway from angiotensinogen to AT1, AT2 and Mas receptors. ACE, Angiotensin-converting enzyme

More components of the RAS such as ACE2, angiotensin-(1–7) and the Mas receptor have recently been identified in the brain. This alternative pathway is sometimes referred to as the non-classical RAS [57]. Originally identified in 1986 as an oncogene in mice [58], the tumorigenic power of Mas was later discredited and remained an orphan receptor until it was subsequently shown to bind with Ang (1–7) [17]. ACE2 can hydrolyse Ang II to produce Ang-(1–7). It can also cleave Ang I, producing Ang-(1–9) with subsequent Ang-(1-7) formation, although with much less efficiency. Mas is thus proposed to be a receptor for Ang-(1-7), with its highest expression in the brain [59]. The action of Ang-(1–7) through Mas is thought to influence arachidonic acid production and nitric oxide synthase activation [60] (see Fig. 2).

The recently discovered PRR is highly expressed in the brain [18]. Its large extracellular domain binds and captures renin and its almost inactive precursor prorenin, increasing their enzymatic activities [61], but it also mimics the actions of AT1R through intracellular signalling [62].

A specific receptor for angiotensin IV (Ang IV), another less active peptide than Ang II, was first identified in a guinea pig hippocampus [19]. It is thought that the identity of AT4R was established when it was discovered that Ang IV is a strong inhibitor of insulin-regulated aminopeptidase (IRAP) [63]. IRAP is responsible for oxytocin degradation and, as demonstrated when an injection of Ang IV abolished the antidepressant effects of oxytocin in mice [64], is apparently required for its mood effects to take place. Yet recently, discrepancies between Ang IV binding site-antagonist and IRAP inhibitors [60], or the unaltered cognitive response of Ang IV in IRAP knockout mice [65], have cast doubt on whether IRAP is the only AT4R receptor. Further candidates for the role of AT4R have been proposed [42].

Ang II is also involved in cerebral blood flow regulation [21, 22]. Rising circulating Ang II is free to cross into the subfornical organ. This is a circumventricular organ lacking the blood–brain barrier, which, via AT1R, signals the paraventricular nucleus of the hypothalamus to activate the rostral ventrolateral medullary neurons and peripheral sympathetic nerves, thereby raising blood pressure [66]. Overstimulation of AT1Rs can lead to endothelial dysfunction [67] and neuronal injury and vulnerability caused by cerebrovascular remodelling [68–72].

It is well established that angiotensin receptors are present in the brain, yet the origin of active angiotensin peptides in the brain remains somewhat controversial. Researchers are puzzled because while Ang II is too hydrophilic to cross the blood–brain barrier [73], expression of renin in the brain is too low to account for its local synthesis [74]. Among the hypotheses advanced to solve this apparent paradox are renin-independent synthesis of angiotensin peptides [75]; impaired blood–brain barrier in hypertension leading to Ang II leaking into the cerebrospinal fluid [73]; an intracellular form of renin in the brain [76] or undetectable renin caused by its sequestration by PRR [62]. Although uncertainties persist, targeting the brain RAS or the peripheral RAS cannot be equal because ACEIs that penetrate the blood–brain barrier are superior to non-centrally acting ones in preventing cognitive decline [77, 78].

## Major depressive disorder (MDD) and neuroinflammation: pre-clinical data

Inflammation is essential for restoring homeostasis in stress, infection and injury [79]. Hormones and circulating pro-inflammatory cytokines, products of neuronal injury and bacterial endotoxins, activate transcription factors. Activated inflammatory cascades with brain parenchymal microglia and blood-derived infiltrating macrophages also participate [80]. A well-regulated central inflammatory chain is fundamental to restore homeostasis, but an exaggerated response can be responsible for chronic inflammation, neuronal damage and a decrease in brain-derived neurotrophic factor [81–86]. Thus, excess or sustained activation of immune responses augments the risk of disease in vulnerable individuals, and can be important in the pathophysiology of many neurological and psychiatric disorders [2, 81, 87–96].

The inflammatory hypothesis [97, 98] postulates that depression is the result of altered immune-inflammatory pathways. This leads to increased immune activation, inflammation, nitro-oxidative stress and alteration of the kynurenine pathway, which ultimately causes changes in monoamine levels. MDD is characterised by a low-grade inflammatory state with increased peripheral levels of inflammatory cytokines, and microglial activation [98–103]. Normalised levels of inflammatory markers are associated with remission of clinical depression [104], while persistently elevated levels are associated with a lack of response to antidepressants [105]. Elevated levels of inflammatory markers such as C-reactive protein (CRP) may increase the risk of a first episode of depression [106, 107]. However, a large Mendelian randomisation study found no causal association between increased CRP levels and depression in people with genetically elevated CRP [108], and also that inflammation may better stratify those who will or will not benefit from anti-inflammatory treatments [109]. More compelling is the strong observation of depressive symptoms induced by interferon-α treatment, both in humans and in animal models [110–113].

Consequently, it has been hypothesised that drugs with anti-inflammatory properties might also demonstrate antidepressant potential. Nonsteroidal anti-inflammatory drugs have shown benefits [114, 115], although no influence was observed in association with antidepressants [116]. Cytokine inhibitors were found to improve depression [117–119] and specific depressive symptoms, such as anxiety [120] and fatigue [117], among patients with psoriasis [117, 118, 120] or ankylosing spondylitis [119]. This finding is supported by evidence from animal models [121]. In an open-label report, aspirin exhibited antidepressive effects, even at low doses [122], and may have a more favourable benefit/risk ratio compared with selective COX-2 inhibitors [123, 124]. Epidemiological reports also support antidepressant effects of aspirin [106, 125]. N-acetylcysteine may also be useful in treating MDD [126–128]. Statins, which apart from their antiatherosclerotic and cardioprotective effects also display neuroprotective and anti-inflammatory effects [129–131], showed the potential to produce mood-related benefits [132] and are associated with a reduced risk of depression [133]. Clinical trials of statins seem to show antidepressant effects in aggregate [134]. In a meta-analysis, supplementing the treatment of severe MDD with polyunsaturated fatty acids (PUFAs) was found to be beneficial, even though its role in mild-to-moderate depression or prevention seems limited [135].

Studies attempting to link depression with genetic variations in the RAS provide additional evidence. Initial reports for the most studied ACE polymorphism (I/D) – the presence or absence of a 287-bp fragment in intron 16 related to ACE serum levels [136] – were inconsistent and a meta-analysis showed no significance [137, 138]. However, other single nucleotide polymorphisms have been associated with depression [139, 140], including the GG genotype of ACE A2350G, which also correlated with higher ACE serum activity [141]. Recently, seven single nucleotide polymorphisms were significantly tied to late-life depression and cortisol levels under stressful circumstances [142]. The AT1R genotype (A1166C) CC is also associated with depression and increased responsiveness to Ang II [6], as well as clinical response [143, 144]. Epigenetic mechanisms also appear to be important, as altered methylation of the regulatory region of the ACE gene has been associated with depression [145]. ACE polymorphisms even seem able to influence antidepressant response [145–147], cognitive function after a depression episode in the elderly [148, 149], or suicide behaviour [150, 151].

The role of aldosterone in depression is an emerging area of research, thus regulation of aldosterone by the RAS is another point to take into account. Patients with primary hyperaldosteronism have depressive symptoms [152, 153]. In animal models, administering aldosterone leads to depressive behaviour [154], anxiety [155] and anhedonia [156]. Eplerone, an aldosterone antagonist, had anxiolytic properties in rats [157]. Poorer clinical outcome in MDD is predicted by higher salivary aldosterone [158, 159]. Conversely, MDD patients with suicidal behaviour had lower concentrations of aldosterone compared to suicidal patients without MDD and non-suicidal depressive patients [160]. Spironolactone, another MR antagonist, induces a sleep pattern characteristic of melancholic depression and reduces the efficacy of amitriptyline [40]. This hints at a non-linear dynamic of aldosterone throughout the MDD episode, prompting its exploration as a biomarker that is able to differentiate depression duration. Indeed, at least in women, higher aldosterone levels are associated with a shorter duration of a depressive episode [159], and in an animal model were used to mark the onset of depression [161].

Taking the above evidence in aggregate, current understanding of the pathophysiology of depression supports the search for novel therapeutics affecting the pathways of inflammation, oxidative biology, apoptosis and neurogenesis. Besides their anti-inflammatory effects, angiotensin receptor blockers (ARBs) and ACEIs have good tolerability, limited side effects and are already widely used drugs approved by the US Food and Drug Administration [162, 163]. Their neuroprotective, anti-inflammatory, vasodilatory [164] and microglia activation inhibitory effects [29] make them candidates for novel therapeutic targets for inflammatory brain diseases and cognitive disorders [21, 29, 30, 165, 166]. In this regard, interesting data is emerging from animal models.

The body of evidence supporting the antidepressant and antianxiety effects of drugs targeting the RAS in animal models is increasing. Mutant mice lacking the angiotensin gene have less depressive-like behaviour in the forced swim test [167]. Pharmacologically decreasing the production of Ang II by administering captopril (an ACEI) produces an analogous result [168].

Blockage of Ang II also leads to antidepressant-like activity in the learned helplessness [169] and chronic mild stress paradigms [170, 171], both more valid models than the forced swim test. Preclinical data also suggests a link between the antidepressant effect and a decrease in Ang II activity; AT1R antagonism by its specific blockers losartan [3], valsartan [171], irbesartan [170] and telmisartan [172] has similar actions to that caused by ACEIs. As with most antidepressants, use of these blockers also seems to have antianxiety properties. Candesartan [21, 173], losartan [174, 175] and captopril [176] reduced anxiety behaviour (promoting exploration) in the elevated plus maze test. Nevertheless, enalapril (a non-centrally acting ACEI) was not effective in normotensive rats [175].

Remarkably, different phenotypes of anxiolytic response to ARBs across different mice strains may be explained by differences in AT1R expression levels [177]. Curiously, mood effects were also apparent in an amphetamine-induced model of mania in mice, which candesartan was able to prevent and treat with comparable efficacy to lithium [30]. Transgenic rats overexpressing Ang-(1-7) [178] or ACE2 [179] showed a reduced anxiety phenotype that is seemingly dependent on Mas signalling, since antagonism of Mas reversed the phenotype. Administering Ang-(1-7) was associated with decreased oxidative stress markers in the amygdala [180]. The same Mas antagonism also prevented the anxiolytic/antidepressant effect of enalapril in transgenic hypertensive rats [181, 182].

These agents seem to influence mood disorders independently of their blood pressure-lowering activity. A study exploring the effect of valsartan in a chronic mild stress model found no change in average blood pressure after a month of treatment, while at the same time registering antianxiety and antidepressant effects [171].

Animal experiments also support the anti-inflammatory and oxidative stress-reducing effects of these drugs as part of their mechanisms of action. Both irbesartan and fluoxetine decreased levels of thiobarbituric-reactive substances – oxidative stress markers – while increasing catalase and glutathione (antioxidants) and serotonin (5-HT) levels in the brain [170]. Valsartan also increased neurogenesis in mice [171]. Captopril and perindopril (both centrally acting ACEIs) [183], telmisartan [183, 184] and candesartan [21, 185, 186] all show anti-inflammatory effects by reducing microglial activation and levels of inflammatory markers such as nitric oxide and TNF-α.

## Clinical data

To date, no RCT has assessed the effects of ACEIs or ARBs in depression. However, observational studies have established a bidirectional link between cardiovascular disorders and depression. Antihypertensive sympatholytic drugs such as reserpine or clonidine can induce depression [187–189], prompting some to propose that sympathetic nervous system hyperreactivity is a common substrate [190, 191]. It was unclear whether this association was caused by hypertension itself, its treatment, or both [192, 193].

A meta-analysis of prospective cohort studies [194] found no evidence that hypertension is a risk factor for depression. However, the contrary – that depression increases the risk of developing hypertension – has been suggested [195] and confirmed by a meta-analysis [196]. In light of all the evidence, the RAS now emerges as a major link between mood and the cardiovascular system.

In the early 1980s, several cases reported that captopril might promote mood elevation in patients with MDD [197–199]. Mood benefits were reported in 9 patients with MDD, and one with bipolar disorder, who were treated with lisinopril (an ACEI) [200]. In each case, patients were being treated for hypertension or cardiac heart failure (see Table 1).

**Table 1:** Summary of clinical evidence

Studies		Findings	Conclusion	Limitations
MDD				
Zubenko et al., 1984	Case report of mood-elevating effect of captopril in three MDD patients	3 patients: 72-year-old man with CHF 44-year-old woman with HT 67-year-old man with CHF	Mood elevation of the 3 cases with captopril	3rd case developed psychotic symptoms
Deicken, 1986	Case report of captopril treatment of MDD	52-year-old man with HT and D	Improvement of MDD symptoms with captopril	
Germain & Chouinard, 1988	Case report of treatment of MDD with captopril	41-year-old man with D and posterior diagnosis of HT	Total remission of the MDD episode with captopril	
Hertzman et al., 2005	Collection of case reports of lisinopril augmenting antidepressant response (9 MDD + 1 BD)	Mood elevation of MDD and stabilised mood of the BD patient with lisonopril in patients already on antidepressants or MSs All patients being treated for HT	Improved mood with a combination of antidepressants and lisinopril	
Rathmann et al., 1999	Case-control study of 972 diabetic patients	OR for MDD: CCB: OR 2.2 (95% CI: 1.2–4.2) BB: OR 2.6 (95% CI: 1.1–7.0) ACEI: OR 1.3 (95% CI: 0.8–2.2)	ACEI associated with reduced risk of MDD	Screening for MDD made by general practitioners
Williams et al., 2016	Case-control study of a 5-year cohort of 961 men with osteoporosis	Exposure to ACEIs yields reduced risk of MDD (OR: 0.15, 95% CI: 0.04–0.51, *P* = 0.003)	ACE inhibitors were associated with a reduced likelihood for MD onset	Recall bias, unrecognised confounding and limited generalisability
Boal et al., 2016	5-year cohort of 144,660 patients	ACEI/ARB: 53% decreased risk of MD admissions CCB & BB: 2-fold increased risk of MD admissions TZ & NT did not attain statistical significance	ACEI/ARB therapy had a neutral effect (or reduced risk) on MDs	Results do not include milder levels of MDs treated in the community
Negative findings in MDD				
Habra et al., 2010	RCT of citalopram in 284 patients with MDD and coronary disease	Use of ACEIs associated with mean HAMD response of 1.36 versus 6.42 for non-ACEI use	ACEI use predicted worse response to antidepressant	Bias for more severe coronary disease
Mood effects in non-depressed population				
Cohen et al., 1984	Case report of mood elation with enalapril	Produced elation in normal volunteers (33% controls and 27% HT subjects)	Mood elation effect	
Croog et al., 1986	RCT on the quality of life of captopril versus methyldopa versus propranolol in 626 male HT patients for 24 weeks	Captopril: fewer side effects, and better scores for work performance, visual–motor functioning, and measures of life satisfaction versus methyldopa (*P* < 0.05 to < 0.01) Captopril: fewer side effects, less sexual dysfunction and greater improvement of measures of general well-being versus propranolol (*P* < 0.05 to < 0.01)	Captopril group had better scores in tests of general well-being	
Testa et al., 1993	RCT on the quality of life of captopril versus enalapril in 379 HT men for 24 weeks	Captopril: more favourable reports of overall quality of life, general perceived health, vitality, health status, sleep, emotional control (*P* < 0.05)	The centrally acting ACEI (captopril) showed superior quality of life reports despite equal anti-HT response	
Johansen et al., 2012	HUNT study (Norway) 55,472 HT patients	OR for depressive symptoms: ACEI: OR 0.54, 95% CI 0.28–1.08 BB: OR 1.20, 95% CI 0.78–1.83 CCBs: OR 1.04, 95% CI 0.70–1.53	Depressive symptoms were reduced in ACEI, compared to BB and CCB group	Self-reported data
Pavlatou et al., 2008	Open-label study of candesartan in 17 diabetic patients for ≥ 3 months	Significant improvement in interpersonal sensitivity (*P* = 0.027) and depression scores (*P* = 0.026)	Candesartan (an ARB) improves affect	No control group
Negative findings in mood effects in non-depressed population				
Callender et al., 1983	Double-blind placebo-controlled crossover trial with captopril in 8 HT patients for 6 weeks	Mood was slightly lower during captopril administration	No evidence of mood effects of captopril during the trial	Small sample and duration of study
Deary et al., 1991	Double-blind crossover trial of atenolol and captopril in 18 HT patients for 12 weeks (6 weeks each drug)	Patients reported feeling less anxious during treatment with atenolol (a BB) (*P* = 0.02).	A BB was superior to an ACEI in self-reported anxiety	BBs are known to have an effect in somatic anxiety
Omvik et al., 1993	RCT on the quality of life of amlodipine versus enalapril in 461 HT patients for 50 weeks	Indices on quality of life were unchanged or increased in both groups	No difference between a CCB and an ACEI in quality of life	
Fletcher et al., 1992	RCT on the quality of life of cilazapril versus atenolol versus nifedipine in 540 HT patients for 6 months	Little difference between quality of life measures in the cilazapril and atenolol groups. Both superior to nifedipine	No significant differences in quality of life observed between an ACEI and a BB during the trial. Both were superior to a CCB.	More nifedipine dropouts (17%) compared with atenolol (8%) and cilazapril (5%)
Weir et al., 1996	RCT on the quality of life of losartan versus nifedipine in 223 HT patients for 12 weeks	No significant differences in quality of life reports between groups	No significant differences in quality of life were observed between an ACEI and a CCB	Nifedipine had significantly more dropouts (12%) than losartan (5%)

Abbreviations: ACEIs, angiotensin-converting enzyme inhibitors; ARBs, angiotensin receptor blockers; BB, beta-blockers; BD, bipolar disorder; CCB, calcium channel blockers; CHF, congestive heart failure; CI, confidence interval; D, depression; HAMD, Hamilton Rating Scale for Depression; HT, hypertensive; MDD, major depressive disordersl; MDs, mood disorders; MS, mood stabilisers; NT, non-treatment group; OR, odds ratio; RCT, randomised clinical trial; TZ, thiaziades

In a case-control study of 972 patients from primary care practices, who had both diabetes and a new diagnosis of depression, those exposed to ACEIs in the last 6 months showed a lower odds ratio for depression (OR 1.3, 95% CI: 0.8–2.2) compared to those exposed to beta-blockers (BBs) (OR 2.6, 95% CI: 1.1–7.0) and calcium channel blockers (CCBs) (OR 2.2, 95% CI: 1.2–4.2) [201]. In a recent population cohort study, ACEIs decreased the incidence of MDD [202]. These results were replicated by Boal *et al.* [203], who examined mood-related hospital admissions of 144,660 patients treated with antihypertensive monotherapy for a five-year follow-up. Interestingly, ACEIs and ARBs were associated with the lowest risk of mood disorder admissions (log-rank *P* = 0.006), while CCBs (hazard ratio (HR) = 2.28, [95% CI 1.13–4.58]; *P* = 0.02) and BBs (HR = 2.11, [95% CI 1.12 –3.98]; *P* = 0.02) were associated with increased risk compared to ACEIs and ARBs. There was no significant difference in patients receiving no antihypertensive medication (HR = 1.63 [95% CI 0.94–2.82]; *P* = 0.08), or those taking thiazide diuretics (HR = 1.56 [95% CI 0.65–3.73]; *P* = 0.32).

However, in the CREATE trial, a randomised placebo-controlled trial of citalopram in 284 coronary heart disease patients with MDD, the use of ACEIs predicted a worse response to citalopram [204]. A possible caveat is that the use of ACEIs may cause bias towards more severe coronary disease, and thus a possible vascular, more refractory type of depression. Another interesting possibility, considering the antidepressant properties of ACEIs, is that their use may have prevented or even treated milder episodes of depression, creating a selection bias for more severe depression. Indeed, we know that an increasingly smaller percentage of patients respond or remit after trying a second or third drug after failing previous treatments [205], and that antidepressant-naïve patients improve their Hamilton Depression Rating Scale score more than those taking antidepressants in response to treatment [206].

The antidepressant effects of ACEIs can be further inferred both by mood effects in the population without a formal diagnosis of MDD, and in studies looking at quality of life. Mood elation was reported in healthy volunteers taking enalapril [207]. One RCT found a higher quality of life score was attained in patients taking captopril compared to other classes of antihypertensive drugs, despite similar blood pressure control [208]. A head-to-head comparison of captopril (a centrally acting ACEI) and enalapril (a non-centrally acting ACEI) reported no difference in antihypertensive efficacy, but that captopril had a superior effect on quality of life measurements [209].

In the Norwegian HUNT study [192], the depressive symptoms of a large population of 55,472 patients with systemic hypertension taking an ACEI were compared with those of patients with untreated systemic hypertension. Results showed an important trend in favour of the depressive symptom-reducing effects of ACEIs, as assessed by the Hospital Anxiety and Depression Rating Scale (OR 0.54, 95% CI 0.28–1.08). Interestingly, those on BBs (OR 1.20, 95% CI 0.78–1.83) or on CCBs (OR 1.04, 95% CI 0.70–1.53) showed no reduction in depressive symptoms compared to the untreated systemic hypertension group. Again, this suggests that the pharmacological benefits of ACEIs and ARBs in depression are independent of their antihypertensive effects. A small open-label trial of 17 type 2 diabetic patients taking candesartan for at least 3 months found that depression scores were improved [210].

Nonetheless, there are a few negative reports of the effects of RAS drugs on mood. A small (n = 8), 6-week, double-blind crossover trial found captopril to have no positive effects on mood [211]. Another study found the BB atenolol superior to captopril for self-reported anxiety [212]. However, BBs are known to affect somatic anxiety, so measuring anxiety might not be an appropriate proxy for mood in this case. In a double-blinded trial of 451 hypertensive patients taking either enalapril or the CCB amlodipine for 38 weeks, no differences were found between the two drugs in terms of quality of life measures [213]. Another 6-month double-blind trial with 540 hypertensive patients showed no superiority of cilazapril (an ACEI) over atenolol (a BB) [214]. Losartan was also not superior to nifedepine (a CCB) in a 12-week randomised double-blind trial with 223 hypertensive patients [215].

## Conclusions

A growing body of evidence suggests a role for the angiotensin system in the pathophysiology of MDD. Drugs targeting the RAS reduce oxidative and inflammatory stress and enhance neurogenesis; all documented pathological markers in depression. Despite the heavy burden of depression, new drug development has been underwhelming. While RCTs providing definitive proof are yet to come, available preclinical and clinical data suggest the potential antidepressant properties of ACEIs and ARBs. The search for novel, effective, safe anti-inflammatory drugs that act centrally in the brain are of fundamental interest. Future clinical trials targeting the brain angiotensin system are necessary to verify the usefulness of these agents in treating depression.
